# Pre-diagnostic DNA methylation in blood leucocytes in cutaneous melanoma; a nested case–control study within the Norwegian Women and Cancer cohort

**DOI:** 10.1038/s41598-022-18585-y

**Published:** 2022-08-20

**Authors:** Christian M. Page, Therese H. Nøst, Vera Djordjilović, Magne Thoresen, Arnoldo Frigessi, Torkjel M. Sandanger, Marit B. Veierød

**Affiliations:** 1 Oslo Centre for Biostatistics and Epidemiology, Division for Research Support, Oslo University Hospital, Oslo, Norway; 2 Department of Mathematics, Faculty of Mathematics and Natural Sciences, University of Oslo, Oslo, Norway; 3 Centre for Fertility and Health, Norwegian Institute of Public Health, Oslo, Norway; 4 Department of Community Medicine, UiT The Arctic University of Norway, Tromsø, Norway; 5 K.G. Jebsen Center for Genetic Epidemiology, Department of Public Health and Nursing, Faculty of Medicine and Health Sciences, Norwegian University of Science and Technology, Trondheim, Norway; 6 Department of Economics, Ca’ Foscari University of Venice, Venice, Italy; 7 Oslo Centre for Biostatistics and Epidemiology, Department of Biostatistics, Institute of Basic Medical Sciences, University of Oslo, Oslo, Norway

**Keywords:** Predictive markers, Melanoma, Epigenomics, DNA methylation

## Abstract

The prognosis of cutaneous melanoma depends on early detection, and good biomarkers for melanoma risk may provide a valuable tool to detect melanoma development at a pre-clinical stage. By studying the epigenetic profile in pre-diagnostic blood samples of melanoma cases and cancer free controls, we aimed to identify DNA methylation sites conferring melanoma risk. DNA methylation was measured at 775,528 CpG sites using the Illumina EPIC array in whole blood in incident melanoma cases (n = 183) and matched cancer-free controls (n = 183) in the Norwegian Women and Cancer cohort. Phenotypic information and ultraviolet radiation exposure were obtained from questionnaires. Epigenome wide association (EWAS) was analyzed in future melanoma cases and controls with conditional logistic regression, with correction for multiple testing using the false discovery rate (FDR). We extended the analysis by including a public data set on melanoma (GSE120878), and combining these different data sets using a version of covariate modulated FDR (AdaPT). The analysis on future melanoma cases and controls did not identify any genome wide significant CpG sites (0.85 ≤ p_adj_ ≤ 0.99). In the restricted AdaPT analysis, 7 CpG sites were suggestive at the FDR level of 0.15. These CpG sites may potentially be used as pre-diagnostic biomarkers of melanoma risk.

## Introduction

Incidence of cutaneous melanoma, the most aggressive and lethal form of skin cancer, continues to increase in fair-skinned populations^1^. Ultraviolet radiation (UVR) exposure is the main risk factor of melanoma^2^. By sequencing lesions in different evolutionary stages, Shain et al.^3^ found strong signature UVR mutations detectable at all stages, from benign lesions to invasive melanoma^3^. In some melanoma patients, the tumour suppressor gene *TP53* has occasionally shown mutations with a UVR-signature^4,5^. Still, the most common mutations found in melanomas are the *BRAF* V600E and V600K mutations^6^ although these mutations do not carry the typical UVR signature^7^. The biological pathway from UVR exposure to melanoma onset is not yet fully explained, and could be due to epigenetic changes after UVR exposure.

Epigenetic modifications, such as DNA methylation (DNAm), have been associated with all cancers, including melanoma^8^. However, to our knowledge, previous studies on the epigenetics of melanoma have mostly been based on post-diagnostic sampling^9^. Epigenetic biomarkers have been proposed for both survival and response to treatment in melanoma patients^10,11^. However, the predictive value of these markers for melanoma risk itself has not been evaluated. Since most melanomas appear without association with a precursor lesion^12^, evaluation of pre-diagnostic markers should be based on samples of skin, blood, or saliva stored in biobanks. The DNA from these mixed tissues such as blood or saliva could be confounded by the composition of the different tissues. Based on epigenetic data, it is possible to estimate the relative contribution from each cell type observed in the tissue mix^13^.

The population-based Norwegian Women and Cancer (NOWAC) cohort study^14^ has been used to study the importance of host factors and UVR exposure in melanoma risk^15–17^, and to identify pre-diagnostic epigenetic markers for lung and breast cancer^18–20^. In a nested case–control study within the NOWAC cohort, we aimed to identify biomarkers of melanoma risk in pre-diagnostic blood samples of melanoma cases and cancer free controls, in an epigenome wide association study (EWAS), as well as a subset EWAS on case specific characteristics. To complement our analyses, EWAS was also performed in an open source data set from an independent study on melanoma^21^ and combined with our results.

## Materials and methods

### Material (NOWAC)

The NOWAC cohort includes over 172,000 women aged 30–70 years at recruitment in 1991–2006 (response 54%)^14^. Information on host characteristics and lifestyle factors was collected through baseline questionnaires and up to two follow-up questionnaires. The NOWAC study has high external validity, with no major selection bias^14,22^. Approximately 50,000 women (46–63 years) constitute the post genome cohort within NOWAC and donated a blood sample at inclusion or at the second follow-up (2003–2006)^23^. By using the unique identity number of Norwegian citizens, NOWAC is linked to the Cancer Registry of Norway (CRN) for follow-up of cancer incidence and vital status. We included all incident melanoma cases (n = 183) with an isolated DNA and RNA sample in the biobank per December 31, 2013, and matched each case with one cancer free control, based on time since blood sampling and year of birth (1943–1947, 1948–1952, 1953–1957). The Norwegian Malignant Melanoma Registry (NMMR) was established under the CRN in 2008, and information on tumour thickness for incident cases since 2008 was obtained from the NMMR. For melanoma cases diagnosed before 2008, information on tumour thickness was extracted manually by the CRN’s experienced melanoma registrars from histopathological reports in the CRN archive^24^.

All participants gave written informed consent and the Medical Ethical Committees of North Norway has approved the NOWAC study, the storage of human biological material, as well as this study (2016/976/REK Nord). All methods in this study were performed in accordance with the relevant ethical guidelines and regulations.

### DNA methylation

Details of the DNAm quality control has been described elsewhere^25^. Briefly, DNA were treated with bi-sulfite and hybridised to the Illumina Infinium MethylationEPIC array according to the manufacturer’s protocol. Background subtraction and control normalization were performed with *minfi* to reduce background noise and dye bias^26^. Type I and Type II probes were normalized using the Beta mixture quantile normalization method from the *wateRmelon* R-package^27^. After quality control, 775 528 CpG probes remained in the data set. White blood cell composition was estimated using the Houseman algorithm^13,28^.

To complement our analysis, we included an open source data set from an independent study by Conway et al.^21^ (hereafter referred to as the GSE120878 study) which compared the epigenetic profiles of melanoma biopsies (n = 89) and nevi biopsies (n = 73), all from suspected melanoma biopsies from different patients using logistic regression. Their DNA methylation data were deposited at the GEO database in April 2019 (accession number GSE120878)^21^. In this study, DNA methylation was measured on the Illumina Infinium HumanMethylation450 BeadChip array and processed with the *minfi* R package.

### Covariates

Baseline variables include age at recruitment, birth cohort and recruitment year. Age at blood sample and time in freezer were recorded. Participants reported, by questionnaire, education (≤ 10, 11–13, ≥ 14 years), smoking (never, former, current smoker), hair color (dark brown/black, brown, blond/yellow, red), freckling after sunbathing (yes, no), and the number of asymmetric nevi > 5 mm on the legs (0, 1, 2–3, 4–6, 7–12, 13–24, ≥ 25; categorized as 0, 1, ≥ 2). On the basis of average ambient ultraviolet radiation^29^, region of residence (latitudes 70°–58°) was categorized as low UVR exposure (north Norway), medium–low (central Norway), medium (southwest Norway), and highest UVR exposure (southeast Norway)^30^. In the baseline and follow-up questionnaires, participants reported history of severe sunburns (never, 1, 2–3, 4–5, ≥ 6 times/year), average number of weeks per year spent on sunbathing vacations (never, 1, 2–3, 4–6, ≥ 7 weeks/year), and average use of an indoor tanning device (never; rarely; 1, 2, or 3–4 times/month; > 1 time/week) in childhood (≤ 9 years), adolescence (10–19 years), and adulthood (> 19 years)^30^. The reported frequencies of sunburns, sunbathing vacations and indoor tanning were transformed and multiplied by the length of each interval for each questionnaire^15^. The participants were then classified into five categories; non-exposed and quartiles. To capture the tail of the distribution, the upper quartile was further divided into two equally sized groups (i.e. six categories in total) as described in Page et al.^25^ Cumulative UVR exposure was constructed by summarizing the categories (i.e. scores 0–5) for indoor tanning and sunbathing vacations^15^. Reproducibility of melanoma risk factors in the NOWAC questionnaire was good (kappa/intraclass correlation coefficient 0.49–0.77) and independent of age and education^31^.

### Statistical methods

Conditional logistic regression was used to study the association between future status of melanoma as the outcome and white blood cell composition as continuous exposure, accounting for time to diagnosis and potential confounders: hair colour, nevi, and UVR exposure. To minimize technical variation and capture unmeasured confounding, we constructed surrogate variables using the *sva* package in R^32–34^. The surrogate variables were constructed as orthogonal decompositions of the residuals after projecting melanoma status on the DNA methylation data matrix^33^. We used conditional logistic regression, with control for the matching variables (age at blood sample and time in freezer) to assess the associations between future melanoma as the outcome and DNA methylation, adjusting for lifetime history of sunburns (as an indicator of severe UVR exposure)^15^, hair colour (the best measure of skin sensitivity to UVR exposure in the NOWAC cohort)^17,35^, and surrogate variables as potential confounders.

To control for multiple testing, we used the false discovery rate (FDR) procedure of Benjamini and Hochberg^36^. The genes annotated to the top 2000 CpG sites in our main model were included in an enrichment analysis, using the *Enricher* web interface^37^.

An EWAS was run using logistic regression without any covariate adjustments in the GSE120878 dataset, and the log p-values included as covariates in an adaptive multiple testing FDR method called AdaPT^38^ when correcting our main model for multiple testing. This method is based on the covariate modulated FDR (cmFDR) proposed by Ferkingstad et al.^39^ which weight the FDR significance by information from the new data set. The combined AdaPT analysis was restricted to CpG sites with a nominal p-value < 10^–10^ (N_CpG_ = 2176) in the GSE120878 EWAS and the respective log p-values from these sites were included as side information in AdaPT, for each CpG, respectively.

A prediction model was trained on the same CpG sites (p-value < 10^–10^, N_CpG_ = 2176) from the GSE120878 data, using a regression and decision tree algorithm^40^ similar to that of Onwuka et al.^41^. The prediction model was applied on the NOWAC data set of incident melanoma cases and controls.

Lastly, we performed an EWAS including only the melanoma cases in linear regressions with log transformed tumour thickness as the outcome, adjusting for lifetime history of sunburns and hair colour.

### Institutional review board statement

The Medical Ethical Committees of North Norway has approved the NOWAC study, the storage of human biological material, as well as this sub-study (2016/976/REK Nord). All methods in this study were performed in accordance with the relevant ethical guidelines and regulations.

### Informed consent statement

All participants gave written informed consent.

## Results

Baseline characteristics of the cases and controls are presented in Table 1. Having higher education, being a non-smoker, having blond/yellow/red hair, freckling when sunbathing, and large asymmetric nevi on the legs were more common in the melanoma cases than in the controls. Compared to controls, melanoma cases reported more UVR exposure: lower proportion living in the region with low ambient UVR, the lower proportion experiencing no sunburns, the higher proportions in the highest categories of sunbathing vacations, and lower proportions in the two lowest categories of indoor tanning and cumulative UVR exposure (Table 1). Mean age at melanoma diagnosis was 60.2 years (range 49–70) and mean time from blood sampling to diagnosis was 4.4 years (range 0–9.7 years) (Table 1). Estimated cell-type proportions were similar in cases and controls (0.09 ≤ p ≤ 0.94) (Supplementary Table S1). None of the white blood cell fractions were significantly differently distributed between the melanoma cases and controls after adjustment for time to diagnosis (0.14 ≤ p_adj_ ≤ 0.56) (Supplementary Table S2).

**Table 1:** Characteristics of the cases and their matched controls.

	Melanoma cases, n = 183	Controls, n = 183
Age at recruitment (years), mean (SD)	48.0 (6.55)	47.9 (6.53)
**Birth cohort, n (%)**		
1943–1947	73 (39.9)	73 (39.9)
1948–1952	70 (38.3)	70 (38.3)
1953–1957	40 (21.9)	40 (21.9)
**Recruitment year, n (%)**		
1991–1992	80 (43.7)	81 (44.3)
1996–1997	29 (15.8)	29 (15.8)
Age at blood sample (years), mean (SD)	55.7 (4.24)	55.7 (4.24)
Time in freezer (years), mean (SD)	11.23 (0.97)	11.21 (0.95)
**Education (years), n (%)**		
≤ 10	43 (23.5)	54 (29.5)
11–13	57 (31.1)	55 (30.1)
≥ 14	78 (42.6)	67 (36.6)
Missing	5 (2.7)	7 (3.8)
**Smoking, n (%)**		
Non-smoker	77 (42.1)	61 (33.3)
Former	71 (38.8)	73 (39.9)
Current	33 (18.0)	46 (25.1)
Missing	2 (1.1)	3 (1.6)
**Hair color, n (%)**		
Black/dark brown	23 (12.6)	33 (18.0)
Brown	53 (29.0)	75 (41.0)
Blond/yellow	82 (44.8)	60 (32.8)
Red	15 (8.2)	5 (2.7)
Missing	10 (5.5)	10 (5.5)
**Freckling when sunbathing, n (%)**		
Yes	78 (42.6)	67 (36.6)
No	103 (56.3)	111 (60.7)
Missing	2 (1.1)	5 (2.7)
**No. of large asymmetric nevi on legs, n (%)**		
0	136 (74.3)	147 (80.3)
1	11 (6.0)	11 (6.0)
≥ 2	20 (10.9)	8 (4.4)
Missing	16 (8.7)	17 (9.3)
**Residential ambient UVR**		
Low (northern Norway)	23 (12.6)	33 (18.0)
Medium–low (central Norway)	27 (14.8)	22 (12.0)
Medium (south-west Norway)	37 (20.2)	26 (14.2)
Highest (south-east Norway)	96 (52.5)	102 (55.7)
Missing	0	0
**Lifetime no. of sunburns**^**a**^		
0	15 (8.2)	24 (13.1)
1–20	39 (21.3)	42 (23.0)
21–30	34 (18.6)	24 (13.1)
31–47	45 (24.6)	45 (24.6)
48–58	26 (14.2)	21 (11.5)
59+	20 (10.9)	20 (10.9)
Missing	4 (2.2)	7 (3.8)
**Lifetime no. of sunbathing vacations**^**a**^		
0	14 (7.7)	14 (7.7)
1–29	45 (24.6)	44 (24.0)
30–62	32 (17.5)	44 (24.0)
63–104	39 (21.3)	41 (22.4)
105–143	21 (11.5)	16 (8.7)
144 +	26 (14.2)	17 (9.3)
Missing	6 (3.3)	7 (3.8)
**Lifetime no. of indoor tanning sessions**^**a**^		
0	46 (25.1)	49 (26.8)
1–14	30 (16.4)	32 (17.5)
15–24	50 (27.3)	38 (20.8)
25–120	25 (13.7)	32 (17.5)
121–418	14 (7.7)	18 (9.8)
419+	17 (9.3)	14 (7.7)
Missing	1 (0.6)	0 (0)
**Cumulative UVR**^**b**^		
0	8 (4.4)	10 (5.5)
1	15 (8.2)	18 (9.8)
2	26 (14.2)	20 (10.9)
3	15 (8.2)	31 (16.9)
4	31 (16.9)	26 (14.2)
5	34 (18.6)	25 (13.7)
6	24 (13.1)	24 (13.1)
7	15 (8.2)	15 (8.2)
8	8 (4.4)	7 (3.8)
9	3 (1.6)	5 (2.7)
10	4 (2.2)	2 (1.1)
Age at diagnosis (years), mean (SD)	60.2 (4.89)	N/A
Time from blood sampling to diagnosis (years), mean (SD)	4.44 (2.53)	N/A
Tumor thickness (mm), median (IQR)	0.9 (0.5,1.5)	N/A
**Tumor thickness (mm), n (%)**		
T1 (≤ 1 mm)	109 (59.6)	N/A
T2 (1.01–2.0)	32 (17.5)	N/A
T3 (2.01–4.0)	22 (12.0)	N/A
T4 (> 4)	6 (3.3)	N/A
Missing	14 (7.7)	N/A

Matched by age at blood sample and time in freezer.

^a^Categorized in six categories: non-exposed and quartiles, with the upper quartile further divided into two equally sized groups.

^b^Sunbathing vacations and indoor tanning.

We did not find any CpGs significantly associated with melanoma risk in the genome-wide analyses (0.85 ≤ p_adj_ ≤ 0.99). The top 10 CpG sites are listed in Table 2. The estimated odds ratios (ORs) genome wide were in equal proportions in both directions, indicating no global loss of methylation. The pathway enrichment analysis of the top 2000 CpGs did not identify any pathways previously reported for melanoma (Supplementary Table S3).

**Table 2:** The top 10 CpG sites in the conditional logistic regression analyses of CpG sites for melanoma cases (n = 183) versus controls (n = 183).

CpG ID	Position	Gene	OR	95% CI	p-value	FDR adj. p-value
cg09396032	16q24.1	GSE1	0.000349	(0.00002, 0.0054)	1.672e−06	0.85
cg05900127	6q23.2	TBPL1	42.52	(10.61, 170.45)	8.709e−06	0.85
cg19014186	17q23.2	NACA2	7.03	(3.33, 14.84)	1.661e−05	0.85
cg27080643	16p13.3	SLX4	8.94	(3.87, 20.65)	1.709e−05	0.85
cg24287218	6p21.32	HLA-DPA1	10.38	(4.20, 25.66)	2.066e−05	0.85
cg10964388	12q22	NTN4	16.28	(5.50, 48.20)	2.248e−05	0.85
cg17459743	11q14.1	RSF1	0.13	(0.06, 0.29)	2.268e−05	0.85
cg21150053	5p14.1	CDH9	9.78	(4.02, 23.78)	2.330e−05	0.85
cg03347745	14q23.3	N/A	8.94	(3.79, 21.06)	2.538e−05	0.85
cg02804047	6p21.32	C6orf10	0.12	(0.05, 0.28)	2.709e−05	0.85

Adjusted for lifetime number of sunburns, hair colour, and the 10 first surrogate variables.

*OR* odds ratio, *CI* confidence interval, *FDR* False discovery rate.

In the combined AdaPT analysis, after adjusting our findings with the log p-value from the GSE120878 EWAS, seven CpG sites from the NOWAC study had an FDR adjusted p-value below 0.15 (Table 3). The distribution of the ORs for these seven CpGs were shifted towards higher risk, with 5/7 CpGs having an OR above 1, as compared to the entire set of OR in our main EWAS, where the OR was in equal proportions in both directions.

**Table 3:** The seven CpG sites rejected at FDR level of 0.15 with *AdaPT* (covariate modulated FDR; cmFDR). p value from the conditional logistic regression analyses and cmFDR adjusted with *AdaPT* using log p-values from the GSE120878 analysis.

CpG ID	Position	Gene	OR	95% CI	p value	cmFDR adj. p-value
cg01904393	12q13.13	SLC4A8^a^	4.90	(1.99,12.08)	3.69e−03	0.15
cg25961816	6q22.31	N/A	3.35	(1.65, 6.93)	5.34e−03	0.15
cg05250768	7p15.2	MIR196B/HOXA9-HOXA10^a^	0.16	(0.06, 0.40)	1.12e−03	0.15
cg18919097	3q26.1	C3orf57	11.25	(2.98,42.45)	2.7e−03	0.15
cg03505866	14q24.1	KIAA0247/SUSD6	3.86	(1.78, 8.31)	4.0e−03	0.15
cg14966782	2q12.3	SH3RF3	2.75	(1.69,4.48)	6.2e−04	0.15
cg00974255	17q12	N/A	0.15	(0.06, 0.36)	5.8e−04	0.15

Adjusted for lifetime number of sunburns, hair colour, and the 10 first surrogate variables.

*OR* odds ratio, *CI* confidence interval, *FDR* False discovery rate, *cmFDR* covariate modulated FDR.

^a^GRCh37/hg19 (nearest gene).

The prediction model trained on the GSE120878 data set, did not predict melanoma status well: while 48% were predicted true positive, 49% were predicted as false positive, with the true negative only 1% and the false negative 2%. We did not find any significant CpG associations in the EWAS analysis of DNAm and tumour thickness in the melanoma cases (0.86 ≤ p_adj_ ≤ 0.99).

## Discussion

We compared DNAm profiles of incident melanoma cases to healthy controls, to identify potential biomarkers for melanoma risk. We did not identify any genome wide significant CpGs related to melanoma risk. However, by combining different data sources, weighing the FDR adjustment, we identified seven potentially differentially methylated CpG sites associated to incident melanoma, all previously associated to melanoma in a case–control study^21^.

Two of the top 10 genes identified in our EWAS (Table 2) have previously been associated to melanoma; *RSF1*^42^ and *NTN4*^43^. However, they have been associated with more advanced stages in melanoma from case only studies and cell lines, and not with melanoma risk. We observed an equal number of effect sizes in both directions while the proportion of hypomethylation was larger in the GSE120878 study (50% vs ~ 57.6%), indicating a global loss of methylation in melanoma biopsies, which was not observed in the pre-diagnostic samples. This indicates that the loss of methylation observed in cancers may be a consequence of the disease, and not its cause. The log-odds was consistently over ten times larger in the study including samples from prevalent cases as compared to this pre-diagnostic study, which is also to be expected, given the differences in sample tissues in the two studies.

None of the top genes found in our primary analysis were associated to melanoma risk in the largest GWAS of melanoma to date^44^, which included almost 37,000 melanoma cases and ten times as many controls. Of the CpGs associated with melanoma risk in Table 3, two are associated to genes *MIR196B* and *SH3RF3*, which have been observed differentially expressed in sun exposed skin, as compared to non-exposed skin^45^. Given the prominent role of UVR exposure in melanoma risk, this is a potentially interesting finding that should be followed up. Further among the genes indicated, the *HOXA9*-*HOXA10* cluster has been observed differentially expressed in multiple cancers^46^, and upregulation of *HOXA9* is related to poor survival in melanoma cases^47^. Since the analysis was informed using findings from a previous case–control study on melanoma, all findings in this analysis have previously been associated with melanoma.

None of the white blood cell fractions were significantly differently distributed between the melanoma cases and controls, even after adjusting for time to diagnosis. This indicated that the cell type composition would not be a confounder for disease status, and was thus not adjusted for in our analysis.

The discovery of pre-diagnostic biomarkers relies on a large number of samples with biological material stored in biobanks, since the future cancer status of each participant is unknown. Biobanks of the size needed for this type of incident sampling, are almost exclusively storing biological samples derived from blood. The use of blood leukocytes may explain some of the poor performance for the multi-CpG prediction for melanoma, which did not predict case status with high accuracy. Additionally, the tissue differences need to be kept in mind when comparing the results between pre-diagnostic blood samples and tissue specific cancer samples. Using results from cancer tissue to inform the FDR correction could help detect cancer like signals in blood samples early on in the disease. Circulating blood leukocytes are constantly in contact with all organ systems in the body, and exposed to the same environment, thus, weak signals from the environmental exposure can often be detected in blood leukocytes^48^. Additionally, pre-clinical tumors are likely to shed DNA fragments in the blood stream, which can influence the DNAm signature in the blood sample, and DNAm isolated from whole blood may then contain weak cancer specific signals.

Being nested in the NOWAC cohort, this study benefits from a large population-based cohort with well documented case information and prospective baseline information on major risk factors for melanoma, such as UVR exposure, but the pre-diagnostic biological material was limited to whole blood and with a limited sample size. The distribution of T categories was not even across the cases, as it can be in a selected clinical sample, but reflective of what is found in the general population (i.e. more T1 than T4 melanomas).

NOWAC is a female only cohort, while GSE120878 included both sexes. Previous cancer studies have included only one sex, either only females^14^ or males^49^. Studies addressing association between UV exposure and melanoma, found no interaction between sun exposure and sex^50^. The GSE120878 data set was balanced with respect to sex ratio, and the p-value for any sex differences between the groups not significant^21^.

The lack of genotype information in the cohort is a limitation. Multiple genetic markers have been found to increase melanoma risk^44^, most notable variants in the CDKN2A gene^51^, however, the consent in NOWAC did not open for genotyping of the participants.

We find that the use of covariate modulated FRD methods, like AdaPT, is a good way of combining our results with public data from a different source.

## Conclusion

No epigenome-wide significant associations to melanoma risk was found, but 7 CpGs identified by combining data and previous knowledge was suggestive of melanoma risk. Future melanoma status was not well predicted in this study, however, using a more targeted tissue, such as skin biopsies could have resulted in more informative epigenetic markers for melanoma risk.

## Supplementary Information

Supplementary Tables.

## Data Availability

The DNA methylation data generated and/or analysed in the current study can be accessed upon reasonable request to the originating cohort. Access will be conditional on adherence to both local and national ethical and security policy. R codes used for the analyses presented in the paper are available upon request.
