# Supplementary material for: Pre-diagnostic DNA methylation in blood leucocytes in cutaneous melanoma; a nested case–control study within the Norwegian Women and Cancer cohort
Source: Sci Rep. 2022 Aug 20;12:14200. doi: 10.1038/s41598-022-18585-y (PMC9392730; doi:10.1038/s41598-022-18585-y)
Supplement: Supplementary file 1 — Supplementary Tables. [file 41598_2022_18585_MOESM1_ESM.docx]

Supplementary Table S1 Mean and standard deviation (SD) of the cell type composition in cases and controls. The p-values were obtained using a t-test.

|  | **Melanoma cases** | | **Controls** | |  |
| --- | --- | --- | --- | --- | --- |
| Cell type | Mean | SD | Mean | SD | p-value |
| CD8^+^ T Cells | 0.055 | 0.044 | 0.056 | 0.045 | 0.844 |
| CD4+ T Cells | 0.189 | 0.062 | 0.183 | 0.068 | 0.385 |
| Natural Killer Cells | 0.090 | 0.054 | 0.089 | 0.050 | 0.772 |
| B Cells | 0.025 | 0.021 | 0.029 | 0.021 | 0.147 |
| Monocytes | 0.081 | 0.023 | 0.085 | 0.027 | 0.091 |
| Granulocytes | 0.458 | 0.094 | 0.458 | 0.099 | 0.944 |

Supplementary Table S2 Odds ratio of each cell type on melanoma, adjusted for time to diagnosis; hair colour, nevi, Lifetime no. of sunburns, and the 10 first surrogate variables. The p-value is adjusted for multiple testing with false discovery rate (FDR), since each cell type is analysed independently of each other.

| **Cell type** | **Odds ratio** | **Standard error** | **p-value** | **FDR adj. p-value** |
| --- | --- | --- | --- | --- |
| CD8^+^ T Cells | 5.53 | 3.95 | 0.161 | 0.304 |
| CD4^+^ T Cells | -2.53 | 4.35 | 0.561 | 0.561 |
| Natural Killer Cells | 2.97 | 3.92 | 0.449 | 0.539 |
| B Cells | -14.46 | 6.42 | 0.024 | 0.146 |
| Monocytes | -9.96 | 5.25 | 0.058 | 0.173 |
| Granulocytes | 6.52 | 5.12 | 0.203 | 0.304 |

Supplementary Table S3 Pathway analysis of top hits for melanoma risk

| **#** | **KEGG Pathway** | **Pathway ID** | **p-value** | **FDR adj. p-value** |
| --- | --- | --- | --- | --- |
| 1 | Toxoplasmosis | tgo05145 | 1.0e-03 | 0.326 |
| 2 | Glutamatergic synapse | hsa04724 | 3.0e-03 | 0.457 |
| 3 | mTOR signaling pathway | hsa04150 | 6.0 e-03 | 0.601 |
| 4 | T cell receptor signaling pathway | hsa04660 | 1.2e-02 | 0.768 |
| 5 | VEGF signaling pathway | hsa04370 | 2.3e-02 | 1.000 |
| 6 | Axon guidance | hsa04360 | 1.0e-02 | 0.773 |
| 7 | Progesterone-mediated oocyte maturation | hsa04914 | 2.4e-02 | 0.912 |
| 8 | Neurotrophin signaling pathway | hsa04722 | 2.2e-02 | 1.000 |
| 9 | Fc gamma R-mediated phagocytosis | hsa04666 | 2.8e-02 | 0.868 |
| 10 | Morphine addiction | hsa05032 | 2.8e-02 | 0.789 |
